# The Incidence and Effect of Adverse Events Due to COVID-19 Vaccines on Breakthrough Infections: Decentralized Observational Study With Underrepresented Groups

**DOI:** 10.2196/41914

**Published:** 2022-11-04

**Authors:** Irene S Gabashvili

**Affiliations:** 1 Aurametrix MEBO Research Miami, FL United States

**Keywords:** COVID-19, COVID-19 vaccines, vaccine adverse events, breakthrough infections, decentralized participatory study, elderly, older individuals, medically underserved populations, aging, elderly population, vaccination, genetic disparity, microbiome disparity, impaired immunity

## Abstract

**Background:**

Despite continuing efforts to improve the inclusion of underserved groups in clinical research, gaps in diversity remain. Participation of special populations is especially important when facing problems of unprecedented complexity such as the COVID-19 pandemic. A better understanding of factors associated with the immune response in diverse populations would advance future preventive and curative approaches.

**Objective:**

The objective of this study was to investigate the factors potentially responsible for adverse events following COVID-19 immunization. The study population included adults from rural areas, transitional countries, and those with medically understudied conditions, across a broad age range.

**Methods:**

The study evolved from peer support networks developed during the COVID-19 pandemic. Participants were recruited digitally through online neighborhood and health communities. Some of the participants volunteered as study investigators assisting with offline recruitment and safety monitoring. Individuals who consented to participate were asked to share their vaccination experiences either using constantly evolving web-based surveys or via one-on-one communication. Inferential statistical analysis to estimate differences between study groups was performed using parametric and nonparametric tests.

**Results:**

Of 1430 participants who shared their vaccination experiences, 648 had outcome measures at their 1.5-year follow-up. Significant differences were found between age groups, types of vaccine adverse events (VAEs), incidences of breakthrough infections, and health conditions linked to the microbiome. Pairwise comparisons showed that VAEs interfering with daily activities were significantly higher in both younger (18-59 years) and older age groups (80-100 years, *P*<.001) than in the 60-79–year age group. Short-term VAEs were associated with lower incidence of breakthrough COVID-19 infections relative to those who reported either minimal or long-term adverse events (*P*<.001). A genetic origin was suggested for some adverse reactions.

**Conclusions:**

The findings of this study demonstrate that vaccine adverse reactions in older individuals are being overlooked, and the incidence of VAEs impairing immunity may be higher than previously perceived. Better preventive measures are needed for all those at risk for life-threatening and long-term adverse events due to vaccination. Supportive community-based studies focusing on these populations could add important data to the current body of knowledge. Further and more comprehensive studies should follow.

**Trial Registration:**

ClinicalTrials.gov NCT04832932; https://clinicaltrials.gov/ct2/show/NCT04832932

**International Registered Report Identifier (IRRID):**

RR2-10.1101/2021.06.28.21256779

## Introduction

### Background

While COVID-19 vaccines are highly effective, they can lead to a range of vaccine adverse events (VAEs). The mechanisms underlying VAEs and their association with vaccination efficacy are not completely clear.

Traditional clinical trials are essential for understanding the safety profiles of new interventions, but many people would not enroll themselves. The COVID-19 Citizen Science study [1] and social media analytics [2,3] address some knowledge gaps, but not social- and age-related barriers to participation. Passive surveillance data [4] are subject to the same limitations and insufficient follow-up. Alternative research strategies are needed to complement the existing evidence base.

### Research Objectives

The idea for this study evolved as minority populations were struggling to make vaccination appointments owing to digital inequality, or seeking answers to questions about their pre-existing conditions, which were not addressed by funded research. A previously reported protocol for an ambispective study [5] hypothesized that the safety profile and immune response to COVID-19 vaccines depend on pre-existing health conditions, metabolism, and microbiomes. The objective of this study was to investigate the factors influencing adverse events following COVID-19 immunization in communities that include underrepresented groups.

## Methods

### Study Population

Participants were recruited from private e-neighborhood networks and health support groups via direct emails and social media posts. Particular efforts were made to recruit populations underrepresented in existing research data sets. Individuals with socially debilitating metabolic body odor (MEBO) [6], including extraoral halitosis, “People Allergic to me,” and trimethylaminuria were invited to contribute to the goals of this study along with their relatives. Support groups for autoimmune disorders, neuropathy, tinnitus, and irritable bowel syndrome were also contacted. To ensure the inclusion of digitally disadvantaged individuals, several volunteers served as study investigators providing face-to-face support. To prevent double-counting, investigators had access to lists of subjects maintained by other investigators in their community but not to personally identifiable information. Cookies were not used to guarantee the anonymity of those sharing information without enrollment. To boost engagement, research insights were periodically communicated via the internet. Data collection was automated using rule-based parsing of emails, alerts from social networks, survey spreadsheets, and group application programming interfaces. An inquiry into medical history was made at initial and follow-up data collections.

### Inclusion Criteria

Inclusion criteria were age≥18 years, intention to get vaccinated, and intended availability throughout the study period. No one was excluded for reasons other than age.

### Outcomes

The study’s primary outcome was the incidence of adverse reactions within 14 days of immunization. The secondary outcome was long-term health conditions and the incidence of breakthrough COVID-19 infections that occurred post vaccination with either a single dose of a COVID-19 vaccine or with 2 or more doses.

### Ethical Considerations

Details of the study procedures (the optional nature of all questions, how the information will be used, the ability to withdraw from research, risks, and benefits) were explained to the participants, and informed e-consent was obtained. Ethics approval for primary and secondary analysis was granted by the institutional review board of MEBO Research (IRB00010169, protocol 20210103MEBO). To ensure participant confidentiality, all their data were coded and stored in a decentralized manner with no individual having complete access to sensitive information. All identifiable data were removed from survey and interview responses. Neither participants nor investigators were compensated.

### Data Analysis

Demographic and clinical characteristics of study groups were compared using the Fisher exact test and relative risk calculations for categorical variables and the Mann-Whitney *U* test for continuous variables. All statistical tests were 2-sided, for which a *P* value of ≤.05 and a 95% CI were used to indicate statistical significance. All analyses were conducted using Python (version 3.10).

## Results

### Participant Characteristics

Participant characteristics are described in Table 1. Of the 1430 vaccinated adults and the 648 participants who were followed up, 51% (n=732 and n=329, respectively) were female. Prevalence of chronic disease was age- and sex-matched between the study cohort and the general population. The age at vaccination ranged from 18 to 119 years. The age group of ≥100 years includes 20 vaccinated semisuper- and supercentenarians (10 men and 10 women) with official social media and Gerontology Wiki accounts [7]. These individuals were added to the study database and followed up from early 2021. Since no information is available about their postimmunization symptoms, this group is not included in the VAE analysis.

**Table 1:** Basic descriptive and inferential statistics of the study population.

Characteristics			Vaccinated (n=1430)									1-year follow up (n=648)									All-cause mortality (n=30)		
			Total (n=1430)		No or minimum VAEs^a^ (n=1113)		VAEs (n=317)		*P* value		Total (n=648)			No COVID-19 infection post vaccination (n=389)		Breakthrough COVID-19 infection (n=259)		*P* value		Mortality (n=30)			*P* value
**Age at receipt of the first dose (years), median (IQR)**																							
	All	62 (40-70)		65 (49-72)		42 (30-61)		<.001		58 (38-70)			61 (47-62)		42 (30-66)		<.001		95 (75-110)			<.001	
	Female	63 (41-70)		65 (49-72)		45 (31-64)		Ref.^b^		59 (40-70)			63 (50-72)		43 (32-65)		Ref.		104 (84-114)			Ref.	
	Male	62 (38-71)		65 (46-72)		40 (27-55)		<.001		56 (35-71)			60 (43-72)		42 (29-67)		.90		91 (75-108)			.07	
**Sex, n (%)**																							
	Female	732 (51)		558 (50)		174 (55)		Ref.		329 (51)			201 (52)		128 (49)		Ref.		12 (40)			Ref.	
	Male	698 (49)		555 (50)		143 (45)		.20		319 (49)			188 (48)		131 (51)		.60		18 (60)			.30	
**Adverse events, n (%)**																							
	Short-term	174 (12)		0 (0)		174 (56)		N/A^c^		93 (15)			77 (20)		16 (6)		<.001		1 (3.5)			.20	
	Long-term	143 (10)		5 (0)		138 (44)		N/A		103 (16)			52 (13)		51 (20)		.20		1 (3.5)			.40	
	No or minimal	1113 (78)		1113 (100)		0 (0)		N/A		450 (69)			260 (67)		187 (74)		Ref.		26 (93)			Ref.	
**Age groups (years), n (%)**																							
	18-29	169 (7)		97 (9)		72 (23)		<.001		85 (13)			29 (7)		56 (22)		<.001		0 (0)			N/A	
	30-39	173 (9)		102 (9)		71 (22)		<.001		85 (13)			34 (9)		51 (21)		<.001		0 (0)			N/A	
	40-49	139 (12)		93 (9)		46 (15)		<.001		81 (13)			47 (12)		34 (13)		.10		0 (0)			N/A	
	50-59	164 (17)		127 (11)		37 (12)		<.001		90 (14)			63 (17)		27 (11)		>.99		0 (0)			N/A	
	60-69	401 (24)		356 (32)		45 (14)		Ref.		135 (21)			93 (24)		42 (16)		Ref.		2 (7)			Ref.	
	70-79	301 (20)		272 (24)		29 (9)		.50		116 (17)			78 (20)		39 (14)		.70		8 (29)			.02	
	80-100	62 (7)		45 (4)		17 (5)		<.001		35 (6)			27 (7)		8 (3)		.90		7 (23)			<.001	
	>100	21 (4)		N/A		N/A		N/A		21 (3)			20 (4)		1 (0)		.06		13 (41)			<.001	

^a^VAE: vaccine adverse event.

^b^Ref.: Reference.

^c^N/A: not applicable.

### Evaluation Outcomes

**Figure 1:**
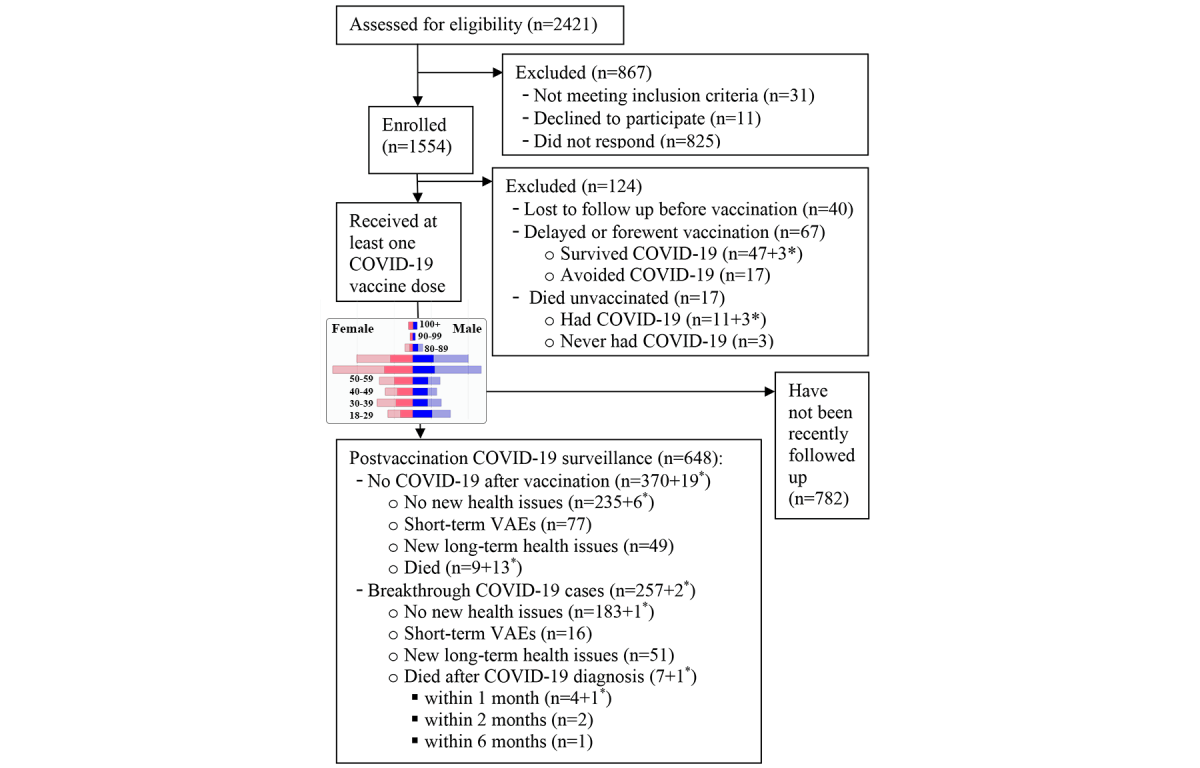
CONSORT (Consolidated Standards of Reporting Trials) flow diagram. Participant flow through the study. Asterisks denote data for the centenarians with publicly available profiles, followed up from early 2021. The population pyramid chart (blue: males; pink: females; transparent shades: no follow-up) shows age and sex distribution of vaccinated individuals (n=1430) and those who were recently followed up (n=648). VAE: vaccine adverse event.

The CONSORT (Consolidated Standards of Reporting Trials) flow diagram in Figure 1 shows the progression of the study, including that of the 1430 subjects who received their first vaccine dose between December 2020 and August 2022 and the 648 individuals whose most recent update was between May and October 2022.

The bar charts in Figure 1 illustrate a balanced representation of both sexes in all age groups, which is also evident in Table 1. Out of 1430 study participants who self-reported outcomes after vaccination, 317 (22%) experienced side effects that prevented them from performing daily activities after receiving at least one of the doses (Table 1). The overall Kruskal-Wallis comparison of VAEs in all age groups was significant (*P*<.001). Pairwise comparisons showed that while the rate of adverse events was similar in the 60-69–year and 70s-79–year age group, the incidence of VAEs in both younger (18-59 years) and older age groups (80-100 years) was significantly higher (*P*<.001).

**Figure 2:**
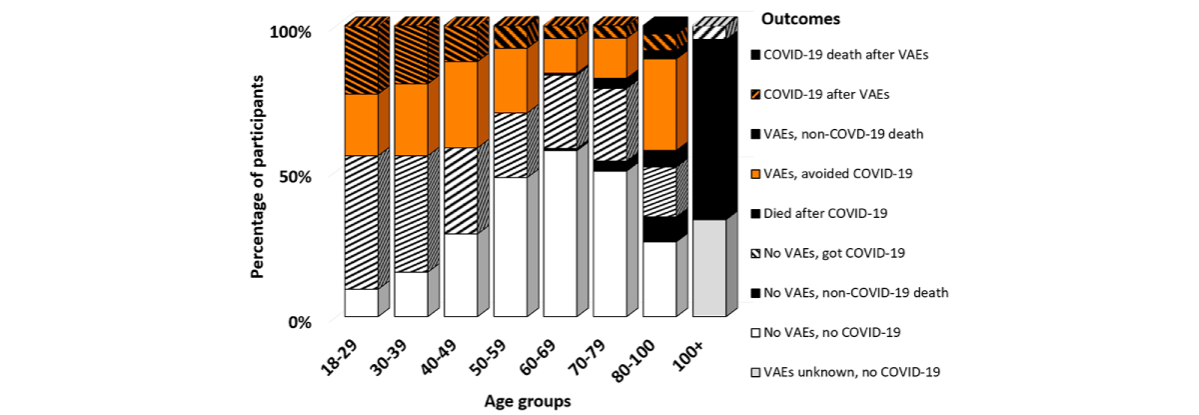
Distribution of participants by adverse event. A white background indicates no or minimal VAEs, while an orange background depicts participants who reported VAEs that prevented them from performing daily activities, diagonal stripes denote breakthrough COVID-19 infection, and solid black represents fatal outcomes. VAE: vaccine adverse event.

Figure 2 displays the distribution of 648 participants by adverse events, or the absence thereof, for each age group. 

**Figure 3:**
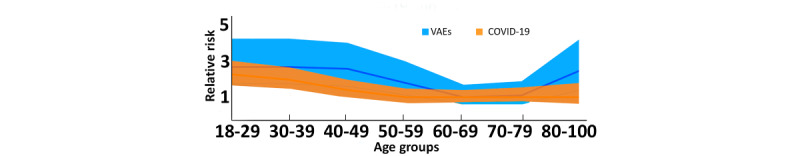
Area charts of CIs for relative risk of vaccine adverse events (VAEs; light blue) vs breakthrough infections (orange) shown for the 648 individuals with recently updated information. Relative risk is shown as a solid line (dark blue: VAEs; orange: COVID-19).

Incidence of breakthrough infections was significantly higher in participants younger than 40 years (Figure 3), as observed previously in many different healthy cohorts [8-10]. 

Another significant association was found between adverse reactions and the ability to avoid infection, but only if symptoms did not last longer than a week (Table 1). We observed higher incidence of pulmonary VAEs in the MEBO [6] subgroup of individuals with halitosis when compared to the group with the second highest prevalence (*P*=.03). We also identified 5 pairs of first-degree relatives with nearly identical sets of VAEs including cardiological events or severe nausea.

Overall, 5%-10% of individuals in chronic disease groups experienced exacerbation of their respective conditions following COVID‐19 immunization. The difference in the incidence of VAEs between healthy individuals and those with chronic conditions was not significant for all age groups (*P*>.05).

There were no significant gender differences in the incidence of VAEs, but age distributions revealed significant differences: a greater risk of VAEs among younger males and higher all-cause mortality in older individuals (Table 1).

## Discussion

### Principal Findings

In summary, we found that adverse events following the COVID-19 immunization are likely to be influenced by a combination of demographic, genetic, and environmental factors. The principal findings of this study were (1) higher incidence of VAEs in both younger and the “oldest old” groups than in “younger old” populations, (2) association of VAEs with immunogenicity observed for short-term but not long-term adverse reactions, and (3) indications that disparities in host genetics and microbiomes in VAEs may exist. 

### Comparison With Prior Work

Age is a known factor contributing to reactogenicity. The heterogeneity is commonly addressed by splitting the sample in 2 groups with the cutoff age between 50 and 65 years [11]. Our study suggests that this simple assumption is not sufficient.

Reporting of VAEs is higher in younger and more educated individuals [12], but it does not appear to translate to higher rates of hospitalization or life-threatening events [13]. We observed a higher incidence of VAEs in both younger and older age groups than in the “young old” populations. We speculate that more adverse events are identified when younger and more educated individuals monitor the oldest participants. The oldest-old participants were more likely to either ignore the side effects or attribute them to aging; for example, duodenal bleeding, markedly decreased appetite [14], and transient amnesia [15] following immunization.

Systemic adverse reactions have been found to be associated with a higher antibody response in mostly healthy younger [16,17] and diseased populations [18,19]. We observed this association across all age groups, health conditions, and genders, but only with respect to short-term effects of vaccination. An impaired humoral immune response was observed after longer-lasting neurological side effects [20,21]. Lower antibody titers were also associated with depressive symptoms after the first and before the second dose of mRNA vaccines [22]. We suggest that more studies are needed on all types of serious and longer-lasting side effects of vaccination.

Host genetic factors are known to contribute to the severity of COVID-19 [23] and stronger short-term reactions to COVID-19 vaccines [24]. Genetic contributions are also being considered for several serious adverse reactions [25,26]. Our preliminary data support the contributions of genetic and microbiome to cardiological and respiratory VAEs. More comprehensive multiomic analyses are needed to draw definite conclusions.

### Limitations

The primary limitation of this study was that the data were obtained from self-reports.

### Conclusions

Our results demonstrate that vaccine adverse reactions in older populations can be easily overlooked. Long-term effects of vaccination in all age groups could outweigh the benefits of this preventive measure in some populations. More research is needed for genetic, epigenetic, metabolome-, and microbiome-associated risk factors of serious VAEs. The prohibitive cost of comprehensive studies [16-24] disproportionally affects underserved populations. Observational trials such as this study therefore represent an effective alternative prescreening strategy for multiomics research.

## Appendix

Multimedia Appendix 1 CONSORT-eHEALTH checklist (V 1.6.1).

## Abbreviations

CONSORT: Consolidated Standards of Reporting Trials
MEBO: metabolic body odor
VAE: vaccine adverse event

## Data Availability

Raw deidentified data and case reports are available upon request to the author, subject to ethics approval.
